# Positive and Negative Symptoms Are Associated with Distinct Effects on Predictive Saccades

**DOI:** 10.3390/brainsci12040418

**Published:** 2022-03-22

**Authors:** Eleanor S. Smith, Trevor J. Crawford

**Affiliations:** 1 Department of Psychology, University of Cambridge, Cambridge CB2 3EB, UK; ess46@cam.ac.uk; 2 Centre for Ageing Research, Department of Psychology, Lancaster University, Lancaster LA1 4YF, UK

**Keywords:** predictive saccades, schizophrenia, bipolar disorder, positive symptoms, negative symptoms

## Abstract

The predictive saccade task is a motor learning paradigm requiring saccades to track a visual target moving in a predictable pattern. Previous research has explored extensively anti-saccade deficits observed across psychosis, but less is known about predictive saccade-related mechanisms. The dataset analysed came from the studies of Crawford et al, published in 1995, where neuroleptically medicated schizophrenia and bipolar affective disorder patients were compared with non-medicated patients and control participants using a predictive saccade paradigm. The participant groups consisted of medicated schizophrenia patients (*n* = 40), non-medicated schizophrenia patients (*n* = 18), medicated bipolar disorder patients (*n* = 14), non-medicated bipolar disorder patients (*n* = 18), and controls (*n* = 31). The current analyses explore relationships between predictive saccades and symptomatology, and the potential interaction of medication. Analyses revealed that the schizophrenia and bipolar disorder diagnostic categories are indistinguishable in patterns of predictive control across several saccadic parameters, supporting a dimensional hypothesis. Once collapsed into predominantly high-/low- negative/positive symptoms, regardless of diagnosis, differences were revealed, with significant hypometria and lower gain in those with more negative symptoms. This illustrates how the presentation of the deficits is homogeneous across diagnosis, but heterogeneous when surveyed by symptomatology; attesting that a diagnostic label is less informative than symptomatology when exploring predictive saccades.

## 1. Introduction

Impairments in eye movements have been recognised in schizophrenia patients since the last century [1], with oculomotor dysfunction identified as one of the most replicated findings in schizophrenia (see [2] for review). These deficits include impaired performance in smooth pursuit [3,4,5] and saccade tasks [6,7], which have been identified both in schizophrenia and the psychosis spectrum surrounding it as well as in the saccade modelling literature [8]. Thus, saccadic paradigms are useful tools in furthering our understanding of the neurophysiological mechanisms involved in oculomotor behaviours in schizophrenia [9,10,11] and other associated neuropsychiatric disorders [12,13,14,15,16].

A crucial human ability that appears to be deficient in multiple neuropsychiatric disorders is the effective calibration of behavioural responses to patterns of motion [14,15,16]. When following an object moving in a predictable pattern, we soon learn to anticipate its movements; thereby establishing a sequence of gaze movements that anticipates the arrival of the target [17]. Bakst and McGuire [18] provide support of oculomotor behaviours in providing a multidimensional interpretation of internal beliefs which display the flexibility in which we utilise and encode information about predictive actions. Hence, when targets are presented in the same locations at fixed times, saccadic initiation occurs not in direct response to the onset of an external stimulus, but rather as part of an implicitly learned sequence [19]. Even without explicit instructions, we readily anticipate these target movements and generate saccades with minimal or even negative latencies, but this has been found to be disrupted in various neuropsychiatric conditions, including schizophrenia [20].

Predictive saccades describe eye movements generated towards an expected target location and their triggering is based on implicitly learned prior knowledge of target behaviour rather than conscious planning [21]. The predictive saccade task is a motor learning paradigm that requires saccades to track the visual target and as the target continues in this predictable pattern, rapid learning ensues, as evidenced by saccade onset times becoming faster than could occur in response to the stimulus appearance. Such anticipatory saccades are therefore generated on the basis of an internalised representation of the visual target [22]. These internally generated anticipatory responses rely on spatial learning and response planning: roles supported by the premotor and dorsolateral prefrontal cortex, hippocampus, and the striatum [23,24].

Crawford et al. [25,26] explored predictive saccades in different neuropsychiatric populations and healthy controls. Crawford et al. [25] found that there were no differences in predictive saccade performance between the bipolar affective disorder (BD), schizophrenia (SZ), and control (CON) groups in latency, gain or final eye position measures (when medication was excluded as an interacting factor). However, when investigating the impact of antipsychotic treatment Crawford et al. [26] reported that non-treated bipolar affective disorder and schizophrenia patients had more accurate saccades than bipolar affective disorder and schizophrenia patients treated with antipsychotics regardless of the targets’ visibility (with regards to saccade gain and final eye position). No differences in the latency of the primary saccades were observed between the groups, but the effect of neuroleptic treatment on the gain measurement was negative, regardless of diagnosis, with neuroleptic treatment associated with significantly lower gain (of the primary saccade and final eye position). The data revealed that medicated patients, whether schizophrenia or bipolar affective disorder, behave similarly, illustrating a lower saccade amplitude, gain control, as well as a greater target undershoot (demonstrated through a final eye position metric) in the neuroleptic-treated patients. The present research aims to explore the influence of symptomatology on saccade gain and final eye position metrics across schizophrenia and bipolar affective disorder patients; controlling for interacting factors such as neuroleptic medication.

With knowledge of the timing of the target’s appearance among neurotypical participants, saccade latencies become anticipatory [27] but only following an adaptation period of ~5-trials [28,29] and with little differences between psychotic groups [25,26]. Among those studies that excluded the first block of trials, focusing only on the stable patterns of predictive saccades, little difference in anticipatory latencies is observed between diagnostic groups [25,26,29,30]. Crawford et al. [25,26] therefore argued that latency may not be a particularly sensitive measure of psychosis. Thus, focusing on the gain and final eye position metrics may be more worthwhile in determining differences between diagnoses on the schizophrenia spectrum, and whether medication plays a significant role in manipulating predictive saccade generation and production.

### Relationship between Prediction Deficits and Schizophrenia Symptoms

In addition to transdiagnostic comparisons, differential saccade performance has also been explored in relation to the dimensional symptomatology of schizophrenia. In general, (medicated) schizophrenic patients performed worse than controls in predictive tasks, but the oculomotor parameters of those with predominantly negative symptoms were found to be different from other patient groups [7]. For example, the latencies of predictive saccades are longer in schizophrenia patients with predominantly negative symptoms, but not predominantly positive or disorganised symptoms, as well as loss of accuracy only being observed as different from controls in this group.

In the antisaccade and smooth pursuit literature, a deficit among schizophrenia patients has been well replicated (see Ettinger et al. [31] for reference); however, the findings of the predictive saccade task are conflicting. Evidence outlined by Obyedkov et al. [7] suggests the importance of exploring the influence of symptomatology, independent of diagnosis, on these potentially endophenotypic deficits. Little research has focused on the symptomatology of psychotic disorders, i.e., the positive and negative symptoms experienced in both schizophrenia and bipolar disorder; the focus has been largely on diagnostic categories. Previous literature reveals that patients with dysfunctional smooth-pursuit eye movements were more likely to experience negative symptoms [32,33,34], but other studies have failed to observe these associations [35,36,37]. Lee et al. [38] conducted a highly informative exploration, highlighting that when schizophrenia patients were considered as a single group, they displayed impairments in smooth pursuit relative to control participants, but a more specific pattern of dysfunction was revealed when distinct symptoms were considered, illustrating a modest relationship with both positive and negative symptoms.

More recent research revealed that individuals with predominantly negative symptoms (taken from a cohort of schizophrenia and chronically high-risk for psychosis (CHR) patients) demonstrated significantly longer latencies in a predictive saccade task than those with predominantly positive symptoms and those in a control group [7]. These results are consistent with previous research finding motor, cognitive, and neuropathological differences in patients with predominantly negative symptoms [39,40]. According to Obyedkov et al. [7], the neural basis for negative symptoms is thought to be deficits in the cortico-striato-thalamo-cortical circuit; predominantly the prefrontal cortex [41,42]. The dorsolateral prefrontal cortex (DLPFC) plays an important role in executive functions—such as working memory, inhibition, and cognitive flexibility [43,44]—which is impaired in psychosis [7]. Given that the DLPFC is also involved in ocular motor behaviour, including intentional saccades [45,46,47,48], dysfunctional processes in individuals displaying predominantly negative symptoms are likely to be observed even in a simple oculomotor task that require executive control. Since predictive saccades rely on a number of executive functions, we hypothesise that those patients who experience predominantly negative symptoms, regardless of their diagnostic status, will manifest a clear impairment.

## 2. Materials and Methods

### 2.1. Participant Selection

The dataset presented in this study came from the Crawford et al. [25,26] studies. The participant groups consisted of medicated schizophrenia patients (M-SZ; *n* = 40; 21 male and 19 female; mean age ± SD = 40 ± 12 years, range = 22–61 years), non-medicated schizophrenia patients (NM-SZ; *n* = 18; 17 male and 1 female; mean age ± SD = 39 ± 13 years, range = 20–61 years), medicated bipolar affective disorder patients (M-BD; *n* = 14; 7 male and 7 female; mean age ± SD = 44 ± 12 years, range = 20–60 years), non-medicated bipolar affective disorder patients (NM-BD; *n* = 18; 12 male and 6 female; mean age ± SD = 42 + 12 years, range = 20–60 years), and controls (CON; *n* = 31; 16 male and 15 female; mean age ± SD = 39 ± 11 years, range = 25–57 years). All patients were identified from the case notes of outpatients at the Royal London Hospital and DSM-III-R criteria [49]. Controls were recruited from among all grades of hospital staff. Informed consent was obtained from all the participants and the study was approved by the Tower Hamlets Ethical Committee. The CONs and their immediate families lacked any history of mental disease. Group matching, exclusion criteria, and further details of the recruitment procedures and clinical assessments are reported in Supplementary Materials (S4) and [25,26]. Group means and standard deviations on all clinical measures are collected in Table 1.

### 2.2. Measurement of Saccades

**Figure 1:**
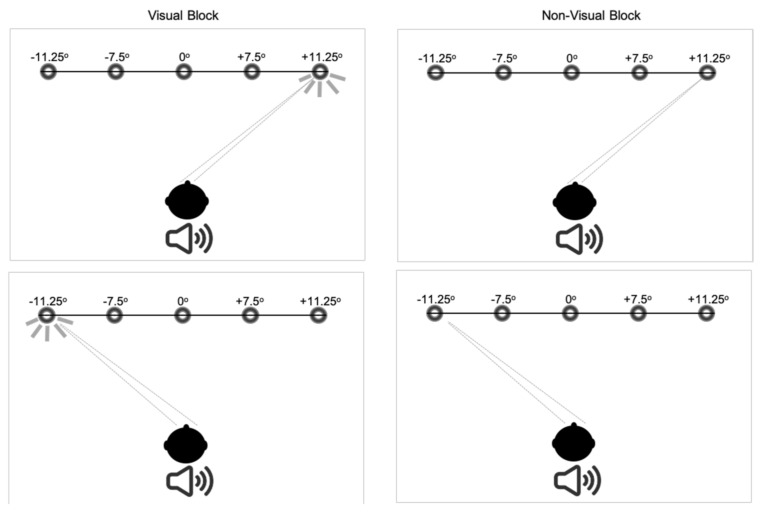
Saccadic target paradigm for the predictive saccade task. See text for full description of the target configuration and the subject instructions in each task.

Apparatus setup, procedure and the predictive saccade paradigm are reported in the Supplementary Materials (S4) and reported in Crawford et al. [25,26] (see Figure 1 for schematic representation of this paradigm).

### 2.3. Data Processing and Statistical Analysis

The first block of predictive trials, in which the regularly alternating targets were visible, showed the usual rapid evolution of latencies towards negative values, as participants developed an anticipatory strategy. Hence, the data from this block were not included in the statistical analyses. In order to explore the effect of target visibility once performance had stabilised, the present research adopted the procedure employed by Crawford et al. [25,26,50]. Here, data from Blocks 2 and 4 were averaged to provide an estimate of performance under non-visual conditions, which was compared to the estimate of performance under visual conditions derived from Block 3. In the present paradigm, the latency and spatial accuracy or gain (i.e., saccade amplitude/target amplitude) of the initial saccade and the gain of the final eye position (FEP) on each trial was analysed. The mean and standard deviation of each parameter was calculated for the two target locations.

## 3. Results

### 3.1. Symptom-Based Analysis

For the symptom-based analyses, a median split was used to divide the participants into a high- and low- negative (determined by their SANS scores) and positive (determined by their SAPS scores) group. Initially, analyses divided positive and negative symptoms by diagnostic category (see Supplementary Materials, S1 for this analysis). Due to unbalanced cohort numbers, individuals experiencing high- and low- levels of negative and positive symptoms were collapsed across diagnosis to explore the singular influence of positive and negative symptomatology (regardless of diagnostic classification) on predictive saccade performance. The final constitution of the groups was as follows: high-negative (*n* = 41: 26 male, 15 female; mean age ± SD = 41 ± 12 years, range = 20–59 years, mean SANS ± SD = 34.4 ± 13.9): low-negative (*n* = 41: 19 male, 22 female; mean age ± SD = 41 ± 12 years, range = 20–61 years, mean SANS ± SD = 4.6 ± 5.5); high-positive (*n*= 40: 24 male, 16 female; mean age ± SD = 38 ± 13 years, range = 20–61 years, mean SAPS ± SD = 24.6 ± 18.9); low-positive (*n* = 42: 21 male, 21 female; mean age ± SD = 43 ± 11 years, range = 20–60 years, mean SAPS ± SD = 0.4 ± 0.9). Note this allows for a participant to be a member of both the negative and the positive classifications. Therefore, additionally, we explored the influence of having negative or positive traits (grouped using a mean ± 0.5 SD split) on performance. See the Supplementary Materials for this analysis (S2).

#### 3.1.1. Saccade Latency

##### Symptom Analysis

**Figure 2:**
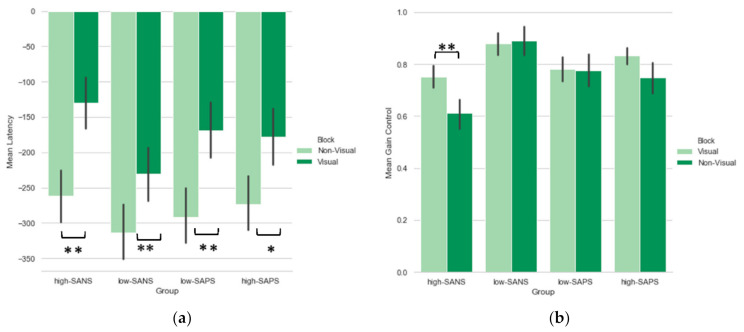

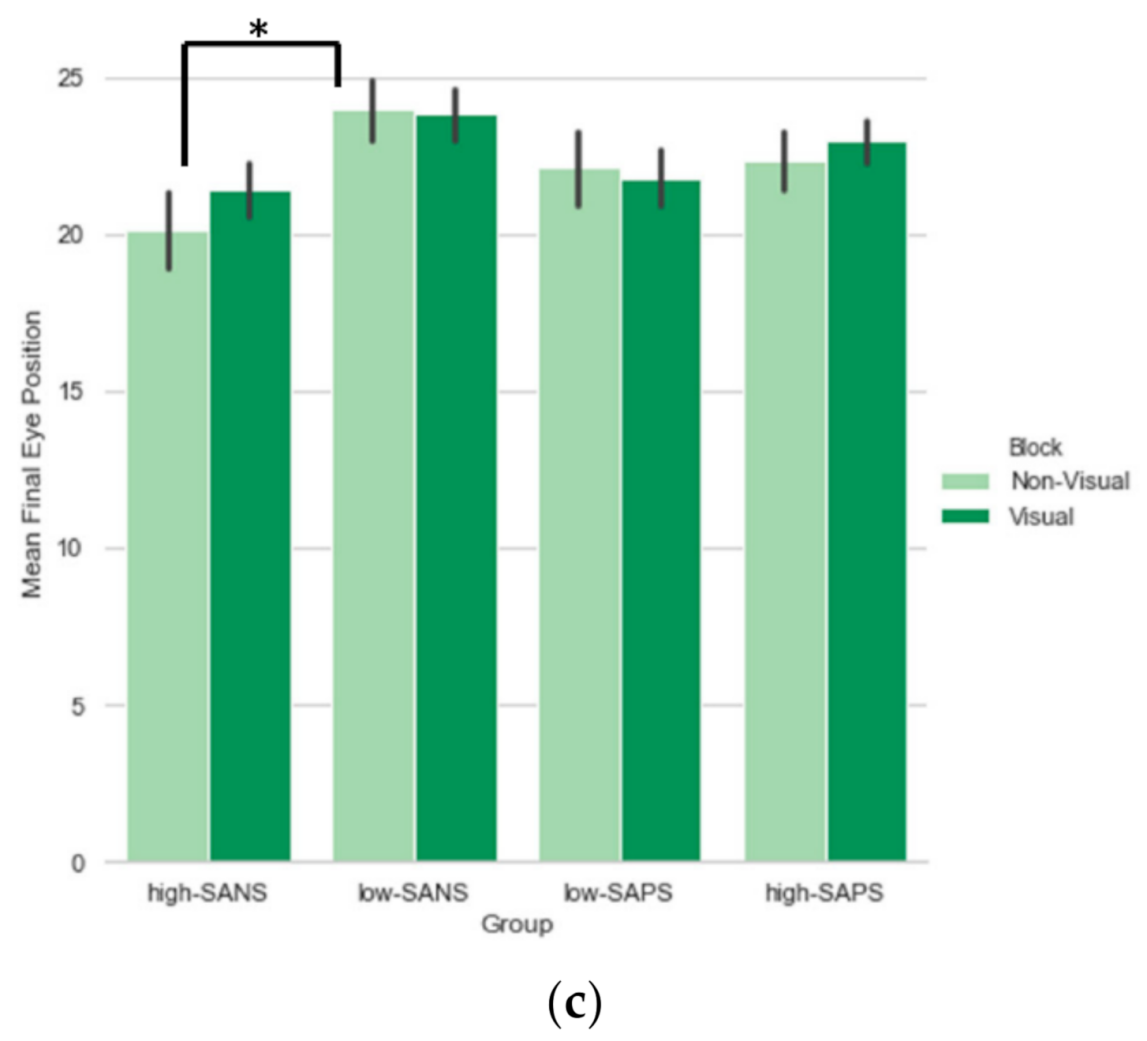
A comparison of performance between the two blocks within each group in the (**a**) latency, (**b**) gain control, and (**c**) final eye position, measure. Error bars refer to standard error. * indicates significance to the *p* < 0.05; ** indicates significance to *p* < 0.01.

A 2 (block: visual, non-visual) x2 (group: high-negative, low-negative) repeated-measures ANOVA with the appropriate Bonferroni corrections was conducted on the latency data. Figure 2a reveals that a significant main effect of visual block condition was evident (F(1,59) = 25.46, *p* < *0*.001, η_p_^2^ = 0.30), with more increased anticipation in the non-visual blocks, but no effect of the symptom group. Post hoc comparisons highlighted latency differences between visual and non-visual blocks in both high-negative (*p* < 0.001) and low-negative (*p* = 0.005) groups. A 2 (block: visual, non-visual) x2 (group: high-positive, low-positive) repeated-measures ANOVA with the appropriate Bonferroni corrections was conducted. A significant main effect of block was observed (F(1,60) = 23.03, *p* < 0.001, η_p_^2^ = 0.28), but no positive symptom group difference (Figure 2a). Post hoc comparisons revealed latency differences between visual and non-visual blocks in both high-positive (*p* = 0.02) and low-positive (*p* < 0.001) groups.

##### Medication Analysis

To explore the effects of medication on symptomatology, a 2 (block: visual, non-visual) × 4 (group: medicated—high-negative, medicated—low-negative, non-medicated—high-negative, non-medicated—low-negative) repeated-measures ANOVA was conducted on the latency data. A main effect of block was again observed (F(1,57) = 17.01, *p* < 0.011, η_p_^2^ = 0.23), but no effect of medication. A 2 (block: visual, non-visual) × 4 (group: medicated—high-positive, medicated—low-positive, non-medicated—high-positive, non-medicated—low-positive) repeated-measures ANOVA was conducted on the latency data. Only a significant effect of block was observed (F(1,58) = 22.27, *p* < 0.001, η_p_^2^ = 0.28).

#### 3.1.2. Saccade Gain

##### Symptom Analysis

Saccade gain refers to the ratio of the saccade amplitude to the target amplitude. A 2 (block: visual, non-visual) × 2 (group: high-negative, low-negative) repeated-measures ANOVA with the appropriate Bonferroni corrections was conducted on the gain data. There was a significant main effect of block (F(1,59) = 3.99, *p* = 0.05, η_p_^2^ = 0.06) and group (F(1,59) = 10.56, *p* = 0.002, η_p_^2^ = 0.15), see Figure 2b. A significant block by group interaction (F(1,59) = 5.63, *p* = 0.021, η_p_^2^ = 0.09) was also observed (Figure 2b). Post hoc comparisons revealed gain control differences between visual and non-visual blocks in only the high-negative (*p* = 0.004) group. A one-way ANOVA was used to explore the group differences; this revealed significantly lower saccade gains in the high-negative group in both the visual (F(1,61) = 4.70, *p* = 0.03) and non-visual (F(1,63) = 12.18, *p* = 0.001) blocks (see Figure 2b). A 2 (block: visual, non-visual) × 2 (group: high-positive, low-positive) repeated-measures ANOVA was conducted, however, no significant differences were found. Clearly a greater deficit is observed among those with high-negative symptoms when contrasted with positive symptoms.

##### Medication Analysis

**Figure 3:**
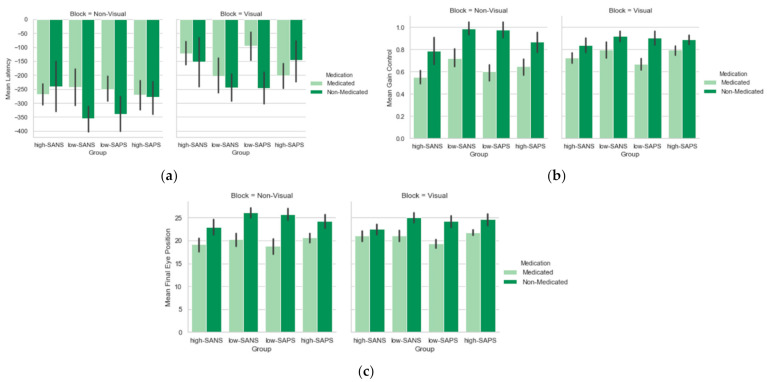
A comparison of the effects of medication on symptomatology between the two blocks in the (**a**) latency, (**b**) gain control, and (**c**) final eye position, measures. Error bars refer to standard error.

To explore the effects of medication on symptomatology, a 2 (block: visual, non-visual) × 4 (group: medicated—high-SANS, medicated—low-SANS, non-medicated—high-SANS, non-medicated—low-SANS) repeated-measures ANOVA with the appropriate Bonferroni corrections was conducted on the gain data. A significant overall effect of medication group was observed (F(3,57) = 6.37, *p* < 0.001, η_p_^2^ = 0.25), as well as a block*medication group interaction (F(3,57) = 2.91, *p* = 0.04, η_p_^2^ = 0.13), but no main effect of block (F(1,57) = 2.74, *p* = 0.10, η_p_^2^ = 0.05). Bonferroni correction for multiple comparisons suggests that the medication group effect was driven by differences between the medicated high-negative and non-medicated low-negative groups (*p* < 0.001). A 2 (block: visual, non-visual) × 4 (group: medicated—high-SAPS, medicated—low-SAPS, non-medicated—high-SAPS, non-medicated—low-SAPS) repeated-measures ANOVA with the appropriate Bonferroni corrections was conducted on the gain data. Figure 3b reveals that only a main effect of medication group was observed (F(3,58) = 5.27, *p* = 0.003, η_p_^2^ = 0.21), with Bonferroni corrections for multiple comparisons suggesting these effects are driven by differences between the medicated and non-medicated low-positive groups (*p* = 0.004), with the medicated group illustrating reduced gain in comparison.

#### 3.1.3. Saccade Final Eye Position

##### Symptom Analysis

The measure of final eye position is important because it provides a ready index of the extent to which the internal representation of the spatial memory is preserved, although this has not been reported in previous studies. A 2 (block: visual, non-visual) × 2 (group: high-negative, low-negative) repeated-measures ANOVA with the appropriate Bonferroni corrections was conducted on the final eye position (FEP) data, where only group differences were observed (F(1,59) = 5.72, *p* = 0.02, η_p_^2^ = 0.09 see Figure 2c). Post hoc comparisons highlighted no significant differences between visual and non-visual blocks in the high- (*p* = 0.08) and low-SANS (*p* = 0.97) group. A one-way ANOVA was used to explore the group differences; illustrating significantly reduced final eye positions in the high-negative groups in the non-visual (F(1,60) = 6.07, *p* = 0.02) block. A 2 (block: visual, non-visual) × 2 (group: high-positive, low-positive) repeated-measures ANOVA with the appropriate Bonferroni corrections was conducted, however, no significant differences were found.

##### Medication Analysis

To explore the effects of medication on symptomatology, a 2 (block: visual, non-visual) × 4 (group: medicated—high-SANS, medicated—low-SANS, non-medicated—high-SANS, non-medicated—low-SANS) repeated-measures ANOVA with the appropriate Bonferroni corrections was conducted on the FEP data. Only a main effect of medication group was observed (F(3,57) = 4.97, *p* = 0.004, η_p_^2^ = 0.21), with Bonferroni corrections for multiple comparisons suggesting these effects are driven by differences between the medicated high-negative and non-medicated low-negative groups (*p* = 0.004), see Figure 3c. A 2 (block: visual, non-visual) × 4 (group: medicated—high-SAPS, medicated—low-SAPS, non-medicated—high-SAPS, non-medicated—low-SAPS) repeated-measures ANOVA with the appropriate Bonferroni corrections was conducted on the FEP data. Only a main effect of medication group was observed (F(3,58) = 4.82, *p* = 0.005, η_p_^2^ = 0.20), with Bonferroni corrections for multiple comparisons suggesting these effects are driven by differences between the medicated low-positive and non-medicated low-positive groups (*p* = 0.007), see Figure 3c.

Overall, the symptom-based analyses reveal differences between the high- and low-negative groups, with significant hypometria and lower gain demonstrated by the high-negative group, which is consistent with the results reported by Obyedkov et al. [7]. The notion that a diagnostic label is not essential in differentiating differences in the predictive saccade paradigm supports the idea proposed by Frith that perhaps symptomatology affords a more complete and sensitive measure of disease deficit.

### 3.2. Symptom Dimensional Analyses

**Figure 4:**
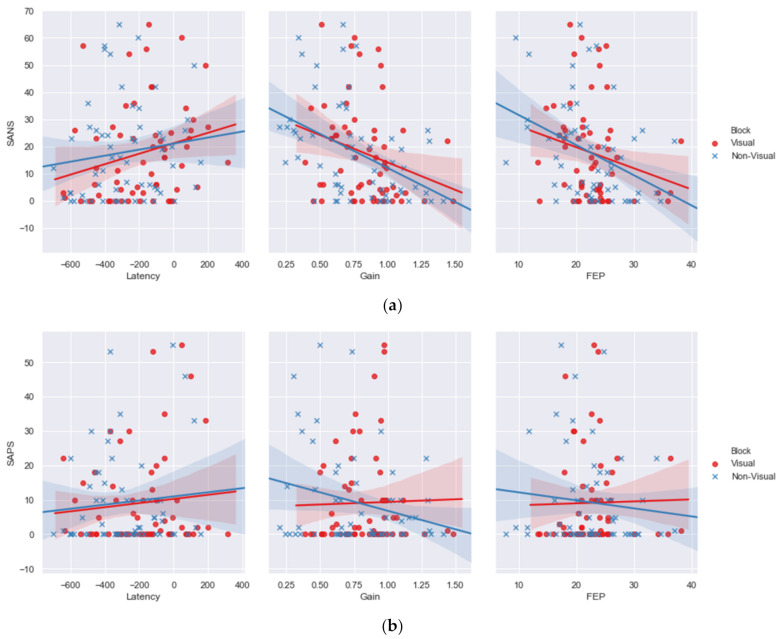
(**a**) Negative correlational relationships were observed between negative symptoms and performance in the visual and non-visual blocks in terms of gain and final eye position measures, and positive relationships between latency and negative symptoms. (**b**) Negative correlational relationships were observed between positive symptoms and performance in the visual and non-visual blocks in terms of gain and final eye position measures.

Pearson correlational analyses explored the relationships between negative and positive symptoms, measured through positive and negative symptoms, and performance in the visual and non-visual blocks of the predictive saccade paradigm. Significant correlational relationships were found between the patients’ SANS scores and the gain measure in the visual (r = −0.266, *p* = 0.04, *n* = 63) and non-visual (r = −0.451, *p* < 0.001, *n* = 65) block (Figure 4a). A negative relationship was also observed between the SANS scores and the final eye position in the non-visual block (r = −0.384, *p* = 0.002, *n* = 62), suggesting that greater hypometria was observed among those with the greatest SANS scores. In Table 1, both groups of schizophrenia patients display more negative symptoms than bipolar affective disorder patients and controls, who systematically display reduced negative symptoms. Thus, these correlations reveal that schizophrenia patients display reduced gain in both the visual and non-visual blocks. Significant relationships were also found between the patients’ SAPS scores and the gain measure in the non-visual (r = −0.274, *p* = 0.03, *n* = 66) block (Figure 4b). No significant correlational relationships were observed for the latency metric.

A proportion of the SANS/SAPS data included in these correlations are equal to 0, which could distort the relationships presented. In order to guarantee the relationships observed in Figure 4a,b are genuine, supplementary analyses can be found in the Supplementary Materials (S3), which were performed on the four diagnostic groups (M-SZ, NM-SZ, M-BD, NM-BD) following the exclusion of those individuals who scored 0 in either the SANS or SAPS measure. These analyses show uniformity with the relational pattern presented in Figure 4a,b.

## 4. Discussion

To our knowledge, this is the first study of predictive saccades to explore the gain control or final eye position metric in relation to psychotic symptomatology. Previous research in this field has relied heavily on the measure of saccadic latency and overlooked other potentially important saccadic parameters. Thus, the present study aimed to provide a more comprehensive analysis of oculomotor deficits, exploring predictive saccade behaviour in terms of latency, gain control, and final eye position. This comprehensive analysis substantially focuses on the approach driven by Frith [51], in which we explore potential “common mechanisms underlying symptoms, which cut across diagnosis” (p11). The present data explore symptom-based analyses, which if Frith’s notions are correct would suggest that the schizophrenia and bipolar disorder diagnostic categories are indistinguishable in their predictive saccade oculomotor patterns, with greater deficits observed among those with predominantly negative symptoms [7].

The current analyses focused on those experiencing predominantly negative or predominantly positive symptoms, highlighting that schizophrenia and bipolar disorder diagnostic categories were indistinguishable in their predictive saccade oculomotor patterns, supporting the dimensional hypothesis of psychosis. No significant group differences were observed when using the latency metric, which contrasts to that reported by Obyedkov et al. [7]; although, when investigated with regard to gain control and final eye position, group differences became apparent among those with predominantly negative symptoms: lower gain in both visual and non-visual blocks, and greater hypometria in the non-visual block. This illustrates how the diagnostic category is not informative in relation to predictive saccades.

### Strengths and Limitations

The current work aimed to provide an update on the existing understanding of the predictive saccade paradigm; taking into account the methodological limitations in the previous literature, and voids that have not been explored previously. In contrast to the majority of published literature on this topic, we included three measures of oculomotor abilities—latency, gain control, and final eye position—to provide a more comprehensive insight into the relationship of psychotic symptoms and prediction. In contrast to the majority of previous studies that have focused on the saccade latency as the primary distinguishing factor in predictive saccade paradigms, the present data support the argument put forward by Crawford and colleagues [25,26], that we should be focusing more on gain control and final eye position as predictors of psychosis severity. A difficulty in drawing conclusions across the literature using the latency metric is driven by inconsistencies in the inclusion of the period of adaptation observed in the first ~5-trials [28,29]. During these first few trials, a drug-naïve schizophrenia group had significantly prolonged latencies compared with the drug-treated schizophrenia group and controls but following this ‘build-up’ no differences in latency were observed. Therefore, in this study the first visual block was excluded to avoid the period of adaptation previously observed in the latency metric [28,29].

Perhaps the heterogeneity exhibited in relation to the predictive saccade task in the existing literature is resultant from a focus on diagnostic category, rather than underlying symptomatology. As far as we are aware there is only one other study that explored the effect of symptomatology on predictive saccade behaviours [7]. Obyedkov et al. [7] reported the need to replicate their findings, considering effects of confounds such as antipsychotic treatment on oculomotor responses to the predictive saccade task. The present analyses incorporated this confound alongside medication status, as well as exploring gain control and final eye position metrics alongside latencies. The existing literature has highlighted a selective relationship between oculomotor deficits and negative symptoms [39,40]. The present work supports this idea, with significantly reduced gain control and greater hypometria among those patients exhibiting predominantly negative symptoms. In further support of this, no significant relationships were found for those with predominantly positive symptoms. Furthermore, saccade gain control differences were also found between medicated and non-medicated patients exhibiting high and low levels of negative symptoms, again with reduced saccade gain control in those with predominantly negative symptoms, which was also mimicked in the final eye position metric. Nevertheless, there is some divergence between the present work and that reported by Obyedkov et al. [7]; firstly, we did not replicate the effect that “latencies of predictive saccades were significantly longer than in controls only in the negative Symptom group” (page 5). Instead, we found no significant main effect of group. This difference may be due to the inclusion of medicated and non-medicated patients in the symptom groups, but when exploring the effects of medication on symptomatology we found no such effect. The difference in latency data may be partially due to the inclusion of the data collected in response to the first experimental block where participants’ latencies are still adapting; highlighting differences in their adaptation process ahead of predictive anticipation.

Classification of the symptomatological features of schizophrenia into positive and negative symptoms has a long history (reviewed by [52,53,54]). In the past it has been suggested that a two-syndrome model is inadequate [55,56] and thus a three-cluster model has been replicated across the literature [57,58,59]. The most consistently replicated of these symptoms clusters, termed psychomotor poverty by Liddle [56], draws similarity with the core negative symptoms [60,61,62,63]. Liddle [64] suggested that psychomotor poverty, and by extension, negative symptoms, may be associated with the malfunction of specific sites in the frontal lobe; reporting associations between psychomotor poverty, an impaired ability to initiate activity, and reduced frontal lobe and basal ganglia functioning [65]. This supports the prior literature describing how in tasks in which participants can use predictable timing information to generate saccade responses, these tasks are thought to make larger demands on timing structures [66,67]. For example, basal ganglia and cerebellar hemispheres are thought to be important to timing ([68], for a review), since damage to either one results in an impairment in the generation of rhythmic movements and the synchronisation of movements with a rhythmic stimulus [50,69], such as in the predictive saccade paradigm. In support of this, Karoumi et al. [28] suggested that the excessive production of anticipatory saccades among schizophrenia patients may be related to difficulties in inhibiting inappropriate saccades (i.e., errors); thus, perhaps more anticipatory saccades are reflective of reduced inhibition and poor performance from the basal ganglia. The neural circuitry involved in the generation of predictive saccades has not been extensively studied in psychosis, but some indication of the important subcortical regions has emerged from studies on Parkinson’s disease. For example, Parkinson’s disease is characterised by a degeneration of the dopaminergic neurons in the nigrostriatal pathway; suggesting the basal ganglia plays an important role in the generation of predictive saccades. The data, however, show mixed results. Research reveals that predictive saccades of Parkinson’s disease patients display a characteristic undershoot of the primary saccades, whereas the final eye positions are no different from those of controls [50,70]. Opposing results in the predictive saccade task have been reported by Gaymard et al. [71] where prolonged latencies were exhibited, and patients were unable to develop predictive behaviour.

In contrast to the strengths of the current research, there are limitations worth noting that can be addressed when moving forward into future predictive saccade explorations. Krebs et al. [30] explored both anticipatory and non-anticipatory saccades; demonstrating that when the saccade target is always visible, group differences between drug-naïve schizophrenia patients and controls were seen in the non-anticipatory saccades, but not for anticipatory saccade latencies. As the present research relied on secondary data analyses it was not possible to explore non-anticipatory saccades, but this should be considered a priori in future work. Another constraint on the present research was the differential sized cohorts; the absence of a sample size calculation for the different groups used throughout the series of hypothesis-driven analyses makes the evaluation of statistical power adequacy difficult. This was highlighted particularly during the symptom-based analyses, where we explored symptomatology—predominantly high-/low-negative and high-/low-positive—within the diagnostic classifications. This analysis can be seen in the Supplementary Materials, but to combat the cohort imbalance high-SANS and low-SANS were collapsed across diagnosis to explore the singular influence of positive and negative symptomatology (regardless of diagnostic classification).

## 5. Conclusions

There is growing and compelling evidence that abnormalities in the voluntary control of eye movements are providing promising endophenotypes for schizophrenia [72,73,74]. Indeed, there has been a remarkable consensus on the prevalence of abnormality in one commonly used paradigm, the antisaccade task, with essentially a 100% replication across multiple studies of the increased error rates in schizophrenia, in comparison to healthy controls. Although this impairment of saccadic eye movements is not restricted to schizophrenia, it is clearly highly perturbed and sensitive to the disease. Given this sensitivity, it is important to consider whether the oculomotor system can help to address the longstanding issue of whether psychotic disorders should be conceptualised as discrete categorical entities, or whether it is more profitable use classifications that are based on the pattern of psychiatric symptoms of the disorder, as Frith and others have proposed. With regards to the current findings using the predictive saccade task, differences in oculomotor behaviour were best captured by the symptom approach, in contrast to a disease classification.

The present data illustrate how the presentation of predictive saccade deficits is homogeneous across diagnosis, but heterogeneous when categorised by symptomatology; clearly, diagnostic labels are not the most informative source of knowledge in relation to predictive saccades. This view is consistent with Frith’s perspective [51] that exploring underlying symptoms is more beneficial than focusing on “essentially arbitrary” (p7) diagnostic definitions. The present data support this idea—the dimensional hypothesis of psychosis—suggesting the deficit presentation is homogeneous; thus, a diagnostic label is not as informative as symptomatology when exploring predictive saccades.

## Figures and Tables

**Table 1:** Clinical, psychiatric, and neuropsychological profile of medicated (M) and non-medicated (NM) schizophrenic and bipolar patients, and control participants, as well as the current dosage of medication outlined in S4 of the Supplementary Materials, expressed in chlorpromazine equivalent units (group means and standard deviation).

	Schizophrenia Patients		Bipolar Disorder Patients		Control Participants
	Medicated	Non-Medicated	Medicated	Non-Medicated	
Chlorpromazine equivalent units	1637 (572)	-	1186 (474)	-	-
Age (year)	39.9 (12.3)	39.4 (13.2)	43.6 (12.1)	42.1 (12.3)	38.51 (10.8)
Disease duration (year)	13.7 (10.7)	12.9 (14.8)	20.5 (11.6)	14.7 (13.6)	-
Age of onset (year)	26.3 (8.9)	25.4 (10.6)	23.1 (8.9)	27.4 (12.2)	-
Negative symptoms (SANS)	27.5 (17.1)	21.5 (19.7)	13.7 (17.6)	4.6 (6.8)	1.0 (1.9)
Positive symptoms (SAPS)	16.6 (21.7)	17.7 (17.8)	5.1 (9.2)	3.4 (6.6)	0.03 (0.18)

## Data Availability

The data will be shared publicly via the Lancaster University PURE system.

## Supplementary Materials

The following supporting information can be downloaded at: https://www.mdpi.com/article/10.3390/brainsci12040418/s1, S1. Summary analyses of the influence of having negative or positive traits (grouped by diagnosis) on performance in the predictive saccade task, S2. Summary analyses of the influence of having negative or positive traits (grouped using a mean ± .5 SD split) on performance in the predictive saccade task, Figure S3. Dimensional supplementary analyses performed on the four diagnostic groups (M-SZ, NM-SZ, M-BD, NM-BD) following the exclusion of those individuals who scored 0 in either the negative symptom or positive symptom measure. These analyses explored the participant’s positive and negative symptoms and their performance in the visual and non-visual blocks of the predictive saccade paradigm in terms of latency, gain and final eye position measures. They show a consistent pattern with those presented in the manuscript, S4. Details of Participant Recruitment, Clinical Measures, Apparatus, Procedure and Predictive Saccade Paradigm, Table S1. Details of the particular neuroleptic drugs taken, as well as the other drug treatments by medicated SZ and BD participants.
