# Supplementary material for: Positive and Negative Symptoms Are Associated with Distinct Effects on Predictive Saccades
Source: Brain Sci. 2022 Mar 22;12(4):418. doi: 10.3390/brainsci12040418 (PMC9025332; doi:10.3390/brainsci12040418)
Supplement: Supplementary file 1 [file brainsci-12-00418-s001.zip › brainsci-1602157-supplementary.pdf]

Supplementary Materials.

S1. Summary analyses of the influence of having negative or positive traits (grouped by diagnosis) on performance in the predictive saccade task.

Initially, exploring the symptomatology of schizophrenia and bipolar disorders in terms of predominantly high- and low- positive/negative scores, the analyses divided these symptom metrics by diagnostic category. However, these analyses had unbalanced cohort numbers: SZ high-negative (*n* SZ= 10), SZ low- negative (*n* SZ = 12), SZ high-positive (*n* SZ = 18), SZ low- positive (*n* SZ = 18), BD high-negative (*n* BD = 6), BD low- negative (*n* BD = 7), BD high- positive (*n* BD = 6), BD low- positive (*n* BD = 20); with greater numbers of positive/negative symptoms among the SZ group, and very few BD individuals identifying as having high positive or negative symptoms.

Saccade Latency

In the first instance, a 2 (Block: Visual, Non-Visual) x4 (Group: SZ - High negative, SZ - Low negative, BD - High negative, BD - Low negative) repeated-measures ANOVA with the appropriate Bonferroni corrections was conducted on the latency data. A significant main effect of block was observed ($F(1,31)=22.60$, $p<.001$, $\eta_p^2=.42$), but no group effect ($F(3,31)=1.38$, $p=.27$, $\eta_p^2=.12$) or interaction was found. A 2 (Block: Visual, Non-Visual) x4 (Group: SZ - High positive, SZ - Low positive, BD - High positive, BD - Low positive) repeated-measures ANOVA with the appropriate Bonferroni corrections was conducted on the latency data. A significant effect of block was observed ($F(1,58)=18.41$, $p<.001$, $\eta_p^2=.24$), but no effect of symptom group or interaction.

Saccade Gain

A 2 (Block: Visual, Non-Visual) x4 (Group: SZ - High negative, SZ - Low negative, BD - High negative, BD - Low negative) repeated-measures ANOVA with the appropriate Bonferroni corrections was conducted on the gain data. A significant main effect of block ($F(1,31)=5.61$, $p=.02$, $\eta_p^2=.15$) and group ($F(3,31)=3.45$, $p=.03$, $\eta_p^2=.25$) was observed. Bonferroni corrections for multiple comparisons highlighted these group differences were driven by differences between the SZ high- negative and the BD low-

negative groups (*p*=.02). A 2 (Block: Visual, Non-Visual) x4 (Group: SZ - High positive, SZ - Low positive, BD - High positive, BD - Low positive) repeated-measures ANOVA with the appropriate Bonferroni corrections was conducted on the gain data. No significant differences between symptom groups or interactions were observed.

Saccade Final Eye Position

A 2 (Block: Visual, Non-Visual) x4 (Group: SZ - High negative, SZ - Low negative, BD - High negative, BD - Low negative) repeated-measures ANOVA with the appropriate Bonferroni corrections was conducted on the FEP data.

No significant differences between symptom groups or interactions were observed. A 2 (Block: Visual, Non-Visual) x4 (Group: SZ - High positive, SZ - Low positive, BD - High positive, BD - Low positive) repeated-measures ANOVA with the appropriate Bonferroni corrections was conducted on the FEP data. No significant differences between symptom groups or interactions were observed.

These results suggest that symptoms are not driving effects within disorders, whilst disorders are affected in a similar manner by block manipulation.

S2. Summary analyses of the influence of having negative or positive traits (grouped using a mean ± .5 SD split) on performance in the predictive saccade task.

Saccade Latency

A 2 (Block: Visual, Non-Visual) x2 (Group: High negative, Low negative) repeated-measures ANOVA with the appropriate Bonferroni corrections was conducted on the latency data. A significant main effect of block was observed ($F(1,19)=24.75$, $p<.001$, $\eta_p^2=.57$), but no group difference was observed. A 2 (Block: Visual, Non-Visual) x2 (Group: High positive, Low positive) repeated-measures ANOVA with the appropriate Bonferroni corrections was conducted. A significant main effect of block was observed ($F(1,25)=24.31$, $p<.001$, $\eta_p^2=.49$), but no group difference was observed.

Saccade Gain

A 2 (Block: Visual, Non-Visual) x2 (Group: High negative Low negative) repeated-measures ANOVA with the appropriate Bonferroni corrections was conducted on the gain data. We observed a significant main effect of block ($F(1,19)=5.32$, $p=.03$, $\eta_p^2=.22$), but no group effect. A 2 (Block: Visual, Non-Visual) x2 (Group: High positive, Low positive) repeated-measures ANOVA with the appropriate Bonferroni corrections was conducted, highlighting a significant effect of block ($F(1,25)=17.22$, $p<.001$, $\eta_p^2=.41$) and a block*group interaction ($F(1,25)=8.44$, $p=.008$, $\eta_p^2=.25$).

Saccade Final Eye Position

A 2 (Block: Visual, Non-Visual) x2 (Group: High negative, Low negative) repeated-measures ANOVA with the appropriate Bonferroni corrections was conducted on the final eye position (FEP) data, where no significant effects were found. A 2 (Block: Visual, Non-Visual) x2 (Group: High positive, Low positive) repeated-measures ANOVA with the appropriate Bonferroni corrections was conducted, where only an effect of block was observed ($F(1,25)=5.39$, $p=.03$, $\eta_p^2=.18$).

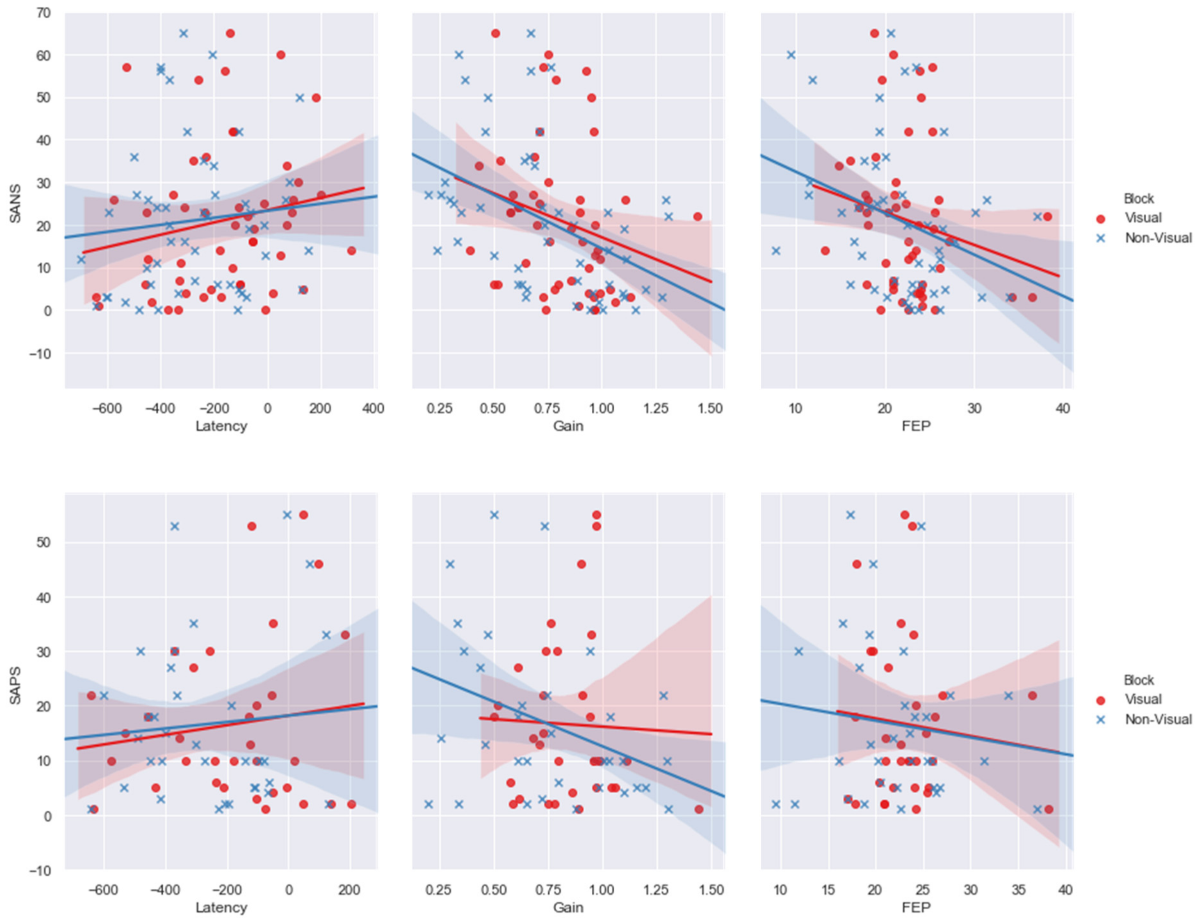

S3. Dimensional supplementary analyses performed on the four diagnostic groups (M-SZ, NM-SZ, M-BD, NM-BD) following the exclusion of those individuals who scored 0 in either the negative symptom or positive symptom measure. These analyses explored the participant's positive and negative symptoms and their performance in the visual and non-visual blocks of the predictive saccade paradigm in terms of latency, gain and final eye position measures. They show a consistent pattern with those presented in the manuscript.

S4. Details of Participant Recruitment, Clinical Measures, Apparatus, Procedure and Predictive Saccade Paradigm.

Participant Recruitment.

Towards the completion of sampling, limited screening on the social, demographic, and cognitive measures was employed to improve the match between the groups. Exclusion criteria included substance abuse, epilepsy, mental handicap, hard neurological signs, visual disturbance other than minor myopia, presbyopia or astigmatism, multiple personality disorder, benzodiazepine use, age outside the range 16-60 years, hearing loss, sever tardive dyskinesia or pathological lying. To be classed as medication-free, a patient must not have received treatment within 6-months prior to the research.

While attempts were made to find patients who were entirely drug free, in the final samples three BD patients were undergoing lithium therapy, two of which were also on carbamazepine, and one was taking antidepressants. Little is known about the effects of such drugs on saccadic eye movements. Iacono et al (1982) in a study comparing unipolar and bipolar affective disorder patients found no significant effects of lithium or antidepressants on reflexive saccade latencies or accuracy, this finding was replicated in a ten-day lithium-placebo randomized double-blind cross-over study with 12 healthy male volunteers (Amado et al., 2005). None of the other subjects was taking drugs thought to affect the central nervous system. Table S1 shows details of the particular neuroleptic drugs taken, as well as the other drug treatment. For the medicated patients, current dosage, expressed in chlorpromazine equivalent units, is shown in Table S1.

Exclusion criteria included: alcohol or substance abuse, dementia, epilepsy, hard neurological signs, major visual dysfunction, multiple personality disorder, age outside the range 18-60, major hearing loss or dyskinesia severe enough to interfere with the experimental tasks. Using hospital records and interviews, it was attempted to match the four groups as closely as possible on independent variables

such as age, time since diagnosis, hospitalization, social class, etc. A successful match was possible on all variables but gender. The male: female ratios were approximately equal for the M-SZ (21:19) and M-BD (7:7) patients, but markedly unequal for the NM-SZ (17:1) and NM-BD (6:12) patients. Particular attention was given to the crucial problem of maximizing the likelihood that the samples of neuroleptic-treated and neuroleptic-free patients were drawn from the same underlying population. The strategy for achieving this in our schizophrenic groups was to concentrate on a set of chronic patients who tended to lapse in attendance at out-patient medication clinics, selecting for our M-SZ group patients whose attendance at 'depo' clinics had shown lapses in the recent past and selecting NM-SZ patients as far as possible from those previously treated but currently lapsed for at least 6 months. This was accomplish satisfactorily for the SZ patients, but such a strategy is inapplicable to BD patients, since many of them are never treated with neuroleptics and those who are treated often have a more severe, and perhaps schizoid, psychosis. At the time of testing most of the BD patients were recovered, exhibiting only the residue of symptoms that might be expected of chronic patients. The mean Best Social Functioning in the last 5 years was significantly poorer overall for the SZ patients (44) than for the BDs (30).

Table S1. Details of the particular neuroleptic drugs taken, as well as the other drug treatments by medicated SZ and BD participants.

	M-SZ	M-BD
Haloperidol	4	4
Flupenthixol deconate	15	1
Trifluoperazinc	5	1
Chlorpromazine	3	5
Fluphcnazine deconate	14	3

Sulpuride	1	0
Clopenthixol deconate	2	2
Others	2	1
Procyclidine/orphenadrine	21	8
Lithium	0	7

Clinical Assessment.

The patients were assessed clinically using a structured interview, which included the Schedule for Affective Disorders and Schizophrenia, (SADS; Spitzer & Endicott, 1975), the Scale for the Assessment of Negative Symptoms (SANS; Andreasen, 1984a), the Scale for the Assessment of Positive Symptoms (SAPS; Andreasen, 19846), and the Mini-Mental State Examination (MM; Folstein et al. 1975). Additionally, their cognitive performance was assessed by the National Adult Reading Test (NART; Nelson, 1982), Raven's Progressive Matrices Test (Raven & Court, 1965). The revised Wisconsin Card Sort test (Heaton, 1981) was used to derive a total error score (WCST); the perseveration score (WCSP) was used as our primary measure of frontal dysfunction and inhibitory control. The presence of tardive dyskinesia (TD) was also reported in order to explore the influence of dopamine in predictive saccade responding – of the M-SZ cohort, 35% reported TD (*n*=14), and of the M-BD cohort, 14.3% reported TD (*n*=2). A full neurological examination was also conducted on each patient. See group means and standard deviations on all measures in Table 1 of the article.

Apparatus.

The target display consisted of 4 red LED targets (diameter 0.25°) ±11.25° either side of the central fixation LED. The LED targets were embedded in a semi-opaque screen and were only visible when illuminated. Subjects were comfortably seated 1.5 m from the screen with a buzzer located centrally behind the subject's head. Movements of the head were constrained by use of an adjustable headrest. The experiment was conducted in the dark. Eye movements were recorded using an infra-red limbus-reflection device (Skalar (IRIS) with a linearity range of ±15°. A hardware antialiasing filter (cut-off frequency 200Hz) was used to filter eye position and the sampling rate was 500 Hz. Blinks were

monitored using electro-oculography with electrodes placed above and below one eye. The stimulus display and data sampling were controlled by a PDP 11/73 computer. Saccadic analysis was conducted off-line using interactive software, which enabled the rejection of artefacts due, for example, to blinks. Saccadic detection was based on a velocity criterion of 30°/s in addition to an acceleration across three consecutive samples. Final eye position was measured by taking the mean fixation location during the maximal period of fixation stability after all secondary corrective saccades were completed.

Procedure.

Each subject participated in four saccade paradigms during the experiment, but the present work will focus only on the predictive saccade paradigm. The predictive saccade block of the paradigm consisted of 44 trials.

Predictive Saccade paradigm.

The participants were informed that the target would alternate horizontally between two fixed locations (±11-25°) at a constant rate of one target step per second (0.5 Hz) and that, in blocks 1 and 3 (vision, V), target onset was synchronous with the onset of a 200ms auditory 'beep' whereas in blocks 2 and 4 (no vision, NV), target visibility would be withdrawn but the buzzer would continue to supply an audible temporal cue. They were instructed to try to maintain the rate and amplitude of saccades in the NV condition. See Figure 1 for schematic representation of this paradigm. Each of the four blocks comprised 11 target steps, though the entire series appeared to the subject continuous.
